# Anti-Citrullinated Protein Antibodies in Patients with Rheumatoid Arthritis: Biological Effects and Mechanisms of Immunopathogenesis

**DOI:** 10.3390/ijms21114015

**Published:** 2020-06-04

**Authors:** Chao-Yi Wu, Huang-Yu Yang, Jenn-Haung Lai

**Affiliations:** 1 Division of Allergy, Asthma, and Rheumatology, Department of Pediatrics, Chang Gung Memorial Hospital, Taoyuan 333, Taiwan; joywucgu@hotmail.com; 2 Chang Gung University, College of Medicine, Taoyuan 333, Taiwan; hyyang01@gmail.com; 3 Department of Nephrology, Chang Gung Memorial Hospital, Taoyuan 333, Taiwan; 4 Division of Allergy, Immunology, and Rheumatology, Department of Internal Medicine, Chang Gung Memorial Hospital, Chang Gung University, Taoyuan 333, Taiwan; 5 Graduate Institute of Medical Science, National Defense Medical Center, Taipei 114, Taiwan

**Keywords:** anti-citrullinated protein antibodies, rheumatoid arthritis, pathogenesis

## Abstract

Individuals with high anti-citrullinated protein antibody (ACPA) titers have an increased risk of developing rheumatoid arthritis (RA). Although our knowledge of the generation and production of ACPAs has continuously advanced during the past decade, our understanding on the pathogenic mechanisms of how ACPAs interact with immune cells to trigger articular inflammation is relatively limited. Citrullination disorders drive the generation and maintenance of ACPAs, with profound clinical significance in patients with RA. The loss of tolerance to citrullinated proteins, however, is essential for ACPAs to exert their pathogenicity. N-linked glycosylation, cross-reactivity and the structural interactions of ACPAs with their citrullinated antigens further direct their biological functions. Although questions remain in the pathogenicity of ACPAs acting as agonists for a receptor-mediated response, immune complex (IC) formation, complement system activation, crystallizable fragment gamma receptor (FcγR) activation, cross-reactivity to joint cartilage and neutrophil extracellular trap (NET)-related mechanisms have all been suggested recently. This paper presents a critical review of the characteristics and possible biological effects and mechanisms of the immunopathogenesis of ACPAs in patients with RA.

## 1. Introduction: Overview of ACPAs in Patients with Rheumatoid Arthritis

Rheumatoid arthritis (RA) is a systemic chronic inflammatory disease characterized by synovial inflammation and joint destruction. It affects 0.5%–1% of the population worldwide, and can conceivably lead to joint destruction and disability [1]. As part of the 2010 American College of Rheumatology/European League Against Rheumatism RA classification criteria, autoantibodies, including rheumatoid factor (RF) and anti-citrullinated peptide antibodies (ACPAs), are hallmarks of the disease [2]. ACPAs, compared to the classic and well-known RA-specific antibody RF, are detected in approximately two-thirds of RA patients, with a superb diagnostic specificity of up to 90% [3,4]. Even though 1%–3% of the healthy populations may also test positive for ACPAs, the levels are mostly in the lower range, and react with a narrow spectrum of citrullinated antigens with different avidities compared to those found in RA [5,6,7,8]. The presence of ACPAs has been shown to precede the onset of clinical symptoms by many years, and is consistently related to a more severe and erosive phenotype [9,10,11,12]. Despite greater disease severity, radiographic joint damage and poorer remission rates [13,14], the presence of ACPAs is also associated with extra-articular manifestations, including pulmonary and cardiovascular involvement [15,16]. Clinically, ACPA status is also relevant for treatment decisions in patients with RA. Accumulating evidence suggests that ACPA seropositivity predicts a good response to rituximab [17,18,19], but is not an ideal biomarker to monitor the efficacy of methotrexate or tumor necrosis factor (TNF) inhibitors [20,21]. Due to their high specificity and excellent clinical correlations, ACPAs have become the autoantibodies of attention in the field of RA research in recent decades [22].

The history of ACPAs can be traced back to 1964, when anti-perinuclear factors were discovered within the sera of RA patients, and later characterized by their reactivity toward citrullinated peptides in 1998 [23]. Since the discovery of ACPAs more than half a century ago, our understanding of their mechanisms of origin and generation has progressed, along with their clinical significance. Recently, the ‘mucosal origin hypothesis’ proposed an environmental trigger in addition to a susceptible genetic background in the development of RA [24]. The revolutionary model for RA development stated that a ‘second hit’ may be required beyond the presence of ACPAs in disease induction [25]. As cigarette smoking and mucosal pathogens are both capable of activating peptidyl arginine deiminase (PAD) and generating citrullinated neoantigens [26,27,28,29], the accumulation of citrullinated antigens and strong immune triggers provided by the virulent agents within the smoke or microbe can possibly attract and activate dendritic cells and B cells for sequential antibody production [25]. With the help of human leukocyte antigen (HLA) molecules, interactions among antigen-specific B cells, T cells and antigen presenting cells (APCs) in the secondary lymphoid organs further promote the maturation of ACPAs [25].

In recent years, our understanding on the generation of ACPAs has progressed significantly, along with the notion of their clinical significance [25,30]. The possible biological effects and pathogenic mechanisms of how ACPAs interact with cells within the synovial space and induce articular inflammation, however, are relatively limited. The joint space among patients with RA is known to be rich with infiltrated immune cells, including lymphocytes, macrophages and neutrophils [1]. The activation of residential fibroblasts and osteoclasts may also contribute to the pathogenesis of RA. Below, we review the latest evidence, discuss several important characteristics of ACPAs, and focus on the interaction between ACPAs and active players in the development of RA.

## 2. Characteristics of ACPAs in RA

Antibodies are Y-shaped proteins produced by plasma cells which recognize unique antigens, and are subdivided into different isotypes according to their crystallizable fragment (Fc) region. The isotype and specificity critically determine an antibody’s immune function and the target it interacts with. While ACPAs and RF are the two leading autoantibodies discovered within patients with seropositive RA and frequently co-occur, these two antibodies exhibit many distinct features and different characteristics which critically determine their roles of pathogenesis. Some distinct characteristics of ACPA, in comparison with RF, are summarized in Table 1, suggesting that the development of these specific autoantibodies from underlying B cell subsets may be through different mechanisms [31]. Compared to IgM-predominant RF, both IgG and IgA are the leading isotypes for ACPAs. Many repeated germinal center reactions, with the help of CD4 T cells, are crucial for the affinity maturation of ACPAs; however, RF-positive B cells only go through a few rounds of germinal center reactions. Furthermore, while all immunoglobulins belong to the glycoprotein family, the extensive N-glycosylation discovered in ACPAs plays an important role in altering immunoglobulin functions. Herein, we discuss the important characteristics of ACPAs which critically dictate their role in the immunopathogenesis of RA.

### 2.1. ACPA Isotypes

The Fc region of ACPAs is crucial in actively contributing to the pathogenesis of RA [32,33]. Unlike the isotypes of RF, where various isotypes (IgM > IgA > IgG) were found [31], the enrichment of IgG and IgA isotypes and an early increase in IgG, particularly IgG1 subclasses, frequency and concentration, were found to dominate in the serum of patients with RA, or to precede the development of the disease [31,34,35,36]. A predominant production of IgA-isotype ACPAs in preclinical samples was also noted among the subjects at risk for RA [37,38]. Interestingly, with its association with the concurrent presence of neutrophils and macrophages, as well as endogenous neutrophil extracellular trap (NET) formation in the sputum, the production of local ACPA IgA was linked to mucosal inflammation [39]. Furthermore, a markedly increased number of IgA-positive plasmablasts, compared to IgG-positive plasmablasts, in at-risk individuals was also observed in comparison with those with early RA and others [40]. IgG is the most important immunoglobulin triggering macrophage activation through Fc gamma receptor (FcγR) engagement, and IgA potently activates neutrophils and is the antibody that dominates in the mucous membranes [41]. The fact that IgG and IgA are the main isotypes of ACPAs in patients with RA not only signals their potential mechanism of action, but also supports the ‘mucosal origin hypothesis’, which has recently been proposed [24].

### 2.2. ACPA Specificity and Avidity

ACPAs consist of a group of antibodies which react with a variety of citrullinated antigens generated through the posttranslational modification of arginine through PADs; namely, citrullination [42,43]. Over the years, various protein candidates for citrullination, such as keratin, fibrinogen, vimentin, fibronectin and α-enolase, have been identified. In fact, the term ‘RA citrullinome’ was introduced recently, referring to the collection of more than 100 citrullinated proteins identified in the serum and synovial fluid of RA patients [44,45,46,47,48]. Although considerable efforts have been devoted by scientists in recent years, no single citrullinated antigen stood out as a dominant epitope for ACPAs [49]. Evidence suggests that it is likely the process of citrullination, rather than any particular citrullinated antigen itself, that drives the autoimmune response in RA [49]. Moreover, as ACPAs are also known to cross-react with peptides undergoing other posttranscriptional modifications, such as carbamylation and acetylation [50,51], the spectrum of epitopes recognized by ACPAs is further extended.

The fine specificity of ACPAs is critical for these antibodies to cause immunopathology. It has been shown that ACPAs isolated from patients with RA not only react with a broad spectrum of citrullinated peptides with different avidities, but also vary from patient to patient with heterogenicity [6,7,8]. Although the broad reactivity of ACPAs may be partially explained by overlapping reactivity, Li and colleagues discovered that, while two-thirds of ACPAs derived from plasmablasts in patients with RA cross-reacted with different tested epitopes, one-third of them recognized only monotargets, including citrullinated fibrinogen, endolase and vimentin [8]. Recently, Ge and colleagues performed a series of crystal structure analyses via X-ray crystallography, and proposed a model to explain the specificity and function of ACPAs [52,53]. As studies demonstrated that several types of structural interactions exist between ACPAs and their citrullinated antigens, the citrulline side-chain-specific ‘promiscuous ACPAs’, the major population of autoantibodies in the sera of RA patients, may not preserve a functional role [52]. On the other hand, the ‘private ACPAs’ interacting with citrulline and proximal amino acid side chains and specifically recognizing or ‘cross-reacting’ with citrullinated epitopes on joint proteins exhibit an arthritogenic role [52]. As an example, through structural studies, Ge and others recently demonstrated that ACPAs could induce the proteoglycan depletion of cartilage and lead to severe arthritis through the cross-reactivity of collagen-type-II-specific ACPAs to joint cartilage [54].

Furthermore, the diversity and avidity of ACPAs toward citrullinated peptides are not fixed, but rather change and evolve with time. Van der Woude and colleagues observed an increase in the amount of citrullinated epitopes recognized by autoantibodies isolated from the sera of pre-RA patients [55]. They showed that the ACPAs isolated from those who were later diagnosed with RA recognized considerably more citrullinated targets than the ACPAs isolated from those who were not [55]. A similar phenomenon was also reported recently by Kongpachith and colleagues, utilizing various citrullinated peptide tetramers [56]. They demonstrated that somatic hypermutations accumulated during affinity maturation by clonally related B cells altered the antibody paratope to mediate ‘epitope spreading’ and the polyreactivity of the ACPA response in RA [56]. In other words, extensive ACPA specificity in RA is derived from a limited repertoire of continuously evolving citrulline-multispecific B cells via constant antigen exposure [57,58,59,60]. In this respect, a vigorous antigen-specific CD4+ T cell response with repetitive germinal center selection would be expected, and indeed has been shown to be the case, for ACPA development soon afterwards [60].

### 2.3. N-Linked Glycosylation of ACPA

N-linked glycosylation is the attachment of glycans or polysaccharides to a protein that is important for both its structure and function [61]. Compared to total IgG, where the glycosylation of the antigen-binding fragment (*Fab*) was estimated to be approximately 14% [62], over 80% of the citrulline-reactive B cells were found to contain glycosylation sites in the sequences of their variable domains [63]. This is consistent with the protein level, where more than 90% of ACPAs were found to carry glycans in their variable domain [64].

The N-linked glycosylation of *Fab* has been shown to be critical in predicting the development of RA [65]. According to Rombouts et al., there was a 10–20 kDa higher molecular mass of the autoreactive ACPAs of RA patients, which resulted from the high frequency of N-glycans in the variable domains [66]. The data indicated that the N-glycosylation sites in ACPA variable domains were introduced by somatic hypermutation, and suggested that ACPA hyperglycosylation confers a selective advantage to ACPA-producing B cells [66]. Similar findings were also supported by other studies suggesting that extensive glycosylation of the IgG ACPA V domain predisposed individuals to the development of RA in a subset of first-degree relatives of indigenous North American RA patients [65]. Interestingly, the sialylation of anti-type II collagen antibodies, including ACPAs, has been found to attenuate arthritogenic activity and suppress the development of arthritis in an experimental murine model [67]. Although Lloyd and colleagues later demonstrated that the sialyation of Fab glycans did not influence antigen binding, the negative charge resulting from sialyation offered selective advantages for ACPA-specific B cells beyond N-linked glycosylation in the variable domain [68]. Together, these data implied not only the importance of N-glycosylation in ACPAs but also the potential pathogenic process directed by the ACPA variable region.

Similar to that of the variable regions, the glycosylation of the Fc fragment is also a unique feature of ACPAs [34,69]. Specifically, antibodies carrying glycans in the Fc portion lack galactose residues and display an enhanced ability to activate the immune system [70,71]. Moreover, in comparison with the pool of serum antibodies, the Fc fragment of ACPAs was generally increased in core fucosylation and decreased in galactosylation and sialylation [34,69]. These glycosylation modifications are important for the molecular interactions and functions of ACPAs [72,73,74]

### 2.4. Synergism between ACPAs and RF through Immune Complex Formation

ICs formed by the conjunction of ACPAs and citrullinated proteins followed by complement and FcγR activation are considered to play a role in ACPA immunopathogenesis [32,33]. Compellingly, when working in conjunction with RF, a synergistic effect is elicited. Partly contributed by the multivalent binding originating from the conformational change in the IgG glycosylation motifs, ACPAs have been documented to work in conjunction with RF to elicit a synergic effect via IC formation and to enhance sequential inflammation [72,73,74]. The synergistic effects of ACPAs and RF in IC formation will be discussed in more detail in the following sections.

## 3. Immunopathogenesis of ACPAs in RA

**Figure 1:**
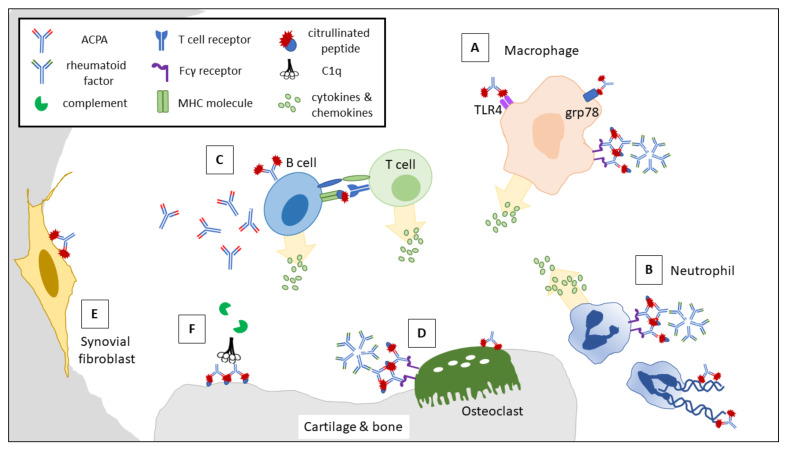
ACPA-related RA immunopathogenesis. ACPAs activate macrophages via IC formation and agonistic activity. They bind to the grp78 protein and TLR4 on the cell surface to elicit an inflammatory response. Additionally, through the binding of ICs and FcγR, proinflammatory cytokines and M-CSF are released (**A**). Neutrophils and ACPAs interact in a self-perpetuating manner. As NETosis releases massive amounts of citrullinated antigens to drive ACPA production, the ACPA formation of ICs promotes further neutrophil netting and the release of degradative enzymes and reactive oxygen species upon binding with FcγR (**B**). Autoreactive B cells baring surface ACPAs can serve as APCs to promote citrulline-specific T cell maturation/differentiation and secrete various proinflammatory cytokines (**C**). Direct ACPA targeting enhances osteoclast differentiation. The binding of ICs via FcγR activates osteoclasts and promotes proinflammatory cytokine production (**D**). ACPAs interact with citrullinated cellular proteins and enhance fibroblast migration (**E**). Complement activation takes place via both the classical pathway and the alternative pathway. ACPAs also cross-react with type II collagen within joint cartilage, resulting in joint destruction (**F**). ACPAs, anti-citrullinated peptide antibodies; RA, rheumatoid arthritis; TLR4, Toll-like receptor 4; FcγR, Fc gamma receptors; IC, immune complex; C5a, complement 5a; M-CSF, macrophage colony-stimulating factor.

Various studies have documented the induction of ACPAs and the arthritogenicity of ACPAs in different murine models [75,76]. The presence of ACPAs in patients with more destructive RA and the accumulation of citrullinated peptides in the rheumatoid joints further suggests the possible role of ACPAs in RA pathogenesis [9,10,11,44,45,46,47,48]. As various immune cells and local tissues are directly involved in the process of joint inflammation, autoantigens are a key sustaining element in autoimmune diseases which fuel the subsequent immune responses. Herein, we approach the issue from the generation of citrullinated antigens and the interactions of ACPAs with RA-related immune players, as well as synovial structural tissues. A summary of the immunopathogenesis of ACPAs within the joint space is depicted in Figure 1.

### 3.1. Dysregulated Citrullination Fuels the Production of Citrullinated Neoantigens for ACPA Targeting

Although citrullinated proteins are known as targets for ACPAs and are potentially active players in RA pathogenesis, citrullination itself is not a pathogenic but a physiological process that is involved in brain development, apoptosis, epidermal differentiation and chromatin modulation [77]. Citrullination is a calcium-dependent process and requires a reducing environment for efficient activity [78,79]. The products from PAD enzymatic reactions have increased protein hydrophobicity and altered protein physical function because of changes in intra- and intermolecular interactions [80,81,82]. However, when excessive citrullination occurs beyond physiological regulation, the resulting citrullinated peptides may potentially become neoantigens to fuel sequential ACPA generation and serve as binding targets [48,83].

The accumulation of intracellular and extracellular citrullinated proteins in rheumatoid joints suggests a citrullination disorder in patients with RA [44,45,46,47,48]. Functional haplotypes of PAD4, one of the enriched PADs discovered in the joints of RA patients [84,85], were also reported to be associated with RA among subjects with different genetic backgrounds [86,87]. In addition, part of the factors, such as environmental stimuli and cell death pathways of various forms, have been suggested to promote the generation of citrullinated autoantigens in RA, as discussed below.

#### 3.1.1. Environmental Factors and ACPA Production

Tobacco smoking and local microbial virulence are the two leading environmental risk factors triggering the generation of ACPA in patients with RA [88,89]. Smoking was also found to be linked to higher PAD2 expression, leading to increased citrullination in patients’ lungs [26]. Such an effect provides an opportunity for the exposure of neo-antigens in patients’ lungs for residential APCs recognition and presentation for sequential antibody production. Next, the strong association of smoking with ACPA positivity in cases with shared epitope (SE) implies a role of CD4 T cells in the RA pathogenesis [90]. Local accumulation of citrullinated antigens and strong immune triggers provided by the virulent agents within the smoke can also possibly attract and activate dendritic cells and B cells for sequential antibody production.

The infection by periodontitis-causing bacteria, such as *Porphyromomas gingivalis* (*P. gingivalis*) and *Aggregatibacter actinomycetemcomitans* (*Aa*), as well as intestinal microbiota, likely contributes to ACPA-associated RA pathogenesis [91,92,93,94,95]. The bacterial pore-forming virulence and calcium ionophores, such as ionomycin and calcimycin from *Streptomyces species* and leukotoxin A from *Aa,* are important in triggering calcium influx and generating non-tolerized neo-citrullinated epitopes [27,28,29]. Citrullination in *P. gingivalis*-related periodontitis works through different mechanisms. *P. gingivalis* is a pathogen capable of citrullinating peptides into a unique pattern due to the expression of its own PAD [96]. However, due to the failure in surveying the actual recognizability of *P. gingivalis* PAD-citrullinated peptides by human ACPA, the definitive conclusions as to periodontitis induced by *P. gingivalis* in ACPA-associated RA pathogenesis cannot be reached according to the currently available data [91]. Interestingly, De Aquino and others have shown that the arthritis induced by *P. gingivalis* is largely dependent on the expansion and activation of Th17, possibly through TLRs and IL-1 [97,98]. Through IL-17Ra signaling, neutrophils infiltrated and aggravated articular inflammation and resulted in local articular destruction [97]. 

#### 3.1.2. Cellular Death Pathways and ACPA Production

The activation of intracellular PADs, the leakage of transiently active PADs into the surroundings and the generation of de novo citrullinated proteins are some of the possible mechanisms related to cellular death pathways [27,99,100,101]. Specifically, autophagy has been shown to lead to the citrullination of α-enolase and vimentin in monocytes and fibroblast-like synoviocytes [100,102]. These citrullinated proteins are known targets for ACPAs, and the level of autophagy markers is well correlated with the titer of ACPAs [100]. Such a notion is also supported by the synchronized reduction of autophagy and levels of citrullinated proteins in patients with RA upon etanercept treatment [103]. In addition, although it is still unclear whether NETosis creates pathogenic citrullination or merely redistributes an existing citrullinome, the release of citrullinated autoantigens extracellularly has been demonstrated to trigger ACPA-associated experimental arthritis [48,99,104]. As previously discussed, bacterial virulence also contributes to cellular damage and results in excessive citrullination [27,28,29]. Collectively, cell death pathways including autophagy, NETosis and a distinct neutrophil death, namely leukotoxic hypercitrullination, have been implicated in the generation of citrullinated auto-antigens and inducing the development of ACPA in susceptible subjects [105].

To date, the significance of the RA citrullinome in the generation of ACPAs and RA pathogenesis has not yet been fully recognized. ‘Exaggerated citrullination of physiologic substrates’ or the ‘citrullination of non-physiological targets’ have been suspected to drive the generation and maintenance of ACPAs. Notably, Andrade and others have found a unique pattern of citrullination over a variety of different proteins in cells isolated from RA patients’ synovial fluid, which they described as hypercitrullination [48]. If nonselective citrullination unwittingly develops due to uncontrolled PAD activity, neo-citrullinated proteins that are not previously tolerized by the immune system may likely trigger an autoimmune response. On the other hand, overactive PADs may target novel sites for reactions and generate neoepitopes by accident. To date, five PADs (PAD1–4 and 6) have been identified in humans. Considering the enrichment of PAD2 and PAD4 in RA synovial tissue and fluid, these two PADs have gained the most attention in RA research [84,85,106]. Previous studies have shown that, compared to PAD4, PAD2 plays a dominant role in fibrinogen citrullination [107]. When catalyzed by PAD4, however, a distinctive and partially citrullinated fibrinogen became preferentially recognized by ACPAs [83]. Collectively, current evidence suggests that dysregulated citrullination may drive the generation of neoantigens for ACPA recognition and targeting in susceptible individuals prone to RA.

### 3.2. Interactions between ACPAs and RA-Related Immune Cells and Mediators

#### 3.2.1. ACPAs Activate Macrophages via IC Formation and Agonistic Activity to Elicit Proinflammatory Cytokine Production

Routinely residing in the synovial space despite inflammation, resting macrophages are potent immune effectors that can robustly promote inflammatory responses and result in joint destruction upon stimulation [108]. ACPAs have been shown to directly interact with surface-expressed citrullinated glucose-regulated protein 78 in RA monocytes with its Fab variable domain [109,110]. Lu and colleagues showed that ACPAs selectively activate extracellular signal-regulated kinases (ERK)1/2 and c-Jun N-terminal kinase (JNK) signaling pathways to activate nuclear factor-kappaB (NF-κB) and promote the production of tumor necrosis factor-alpha (TNF-α) upon binding [109,110]. Compellingly, despite the direct targeting of ACPAs to citrullinated surface protein on monocytes through an unknown interaction, a follow-up study from the same research group documented that ACPAs are also capable of suppressing let-7a, a microRNA, expression in monocytes from RA patients and facilitating inflammatory responses through the JNK and NF-κB pathways [111].

Recently, Sokolove and others have shown with murine and human macrophages that ACPAs against citrullinated fibrinogen elicited higher TNF production than native antibodies [32]. They discovered that ACPAs alone, specifically anti-citrullinated fibrinogen, stimulate macrophages in a Toll-like receptor (TLR)4- and MyD88-dependent manner [32]. Alternatively, when ACPAs were incorporated into the IC, they stimulated macrophages via dual engagement of TLR4/MyD88 and FcγR for the production of TNF-α [32]. Precisely, the binding of ICs to FcγRs on macrophages was not via FcγRI or FcγRIII [112]. Documented by Clavel and others, their data suggested that the cross-linking of FcγRIIa on macrophages upon IC binding strongly activated the signal for cytokine release [112].

Regardless of the diverse interactions, in the presence of macrophage colony-stimulating factor and RF, particularly IgM and IgA isotypes, the ability of ICs to produce proinflammatory cytokines in the synovial membrane appears to be greatly enhanced [72,73,74,113]. The robust secretion of proinflammatory cytokines, particularly TNF-α, by macrophages upon activation strongly promotes inflammatory responses and results in joint destruction [108]. In detail, TNF-α works as an autocrine stimulator for myeloid cells, and serves as a potent paracrine inducer for the production of proinflammatory cytokines critical for lymphocyte proliferation and differentiation [114]. It induces the synthesis of collagenase and prostaglandins, as well as IL-8, by synovial fibroblasts to promote joint cartilage degradation and stimulate more effector cell migration into the synovial spaces [114]. Additionally, its ability to stimulate the expression of endothelial-cell adhesion molecules, suppress regulatory T cells, promote angiogenesis and induce pain sensation are also important mechanisms related to the pathological profiling of RA [108,114].

#### 3.2.2. ACPA-Forming ICs Promote NETosis and the Release of Degradative Enzymes and Reactive Oxygen Species upon Binding with FcγR

The ICs formed within the synovial space and those deposited on the cartilage articular surface were important mediators for neutrophil activation. As defects of FcγRs largely attenuate neutrophil activation in the presence of ICs, similar but different from those of macrophages, the engagement of ICs with FcγRs, particularly FcγRI and FcγRIII, has been documented to trigger neutrophil activation and tissue inflammation [115,116,117,118]. Indeed, recent work put together by Kempers and others documented that ICs containing ACPA IgG1 predominately bind to activating FcγRI and FcγRIIIa. Perhaps because of the enhanced core fucosylation in ACPA IgG1 Fc, ICs bind better to FcγRI expressed by activated neutrophils and drive disease pathogenesis in RA [119]. Specifically, following neutrophil activation, local cartilage and tissue destruction can be induced via neutrophil degranulation and the release of degradative enzymes, as well as reactive oxygen species (ROS) [120]. The activation of soluble receptors and cytokines, the inhibition of chondrocyte proliferation and the activation of synoviocyte proliferation and invasion also contribute to joint injury [120]. The generation of chemoattractants, such as IL-8 and leukotriene B4, further amplify the inflammatory process by recruiting more neutrophils to the synovium [120]. These specific characteristics of neutrophils upon activation possess a great cytotoxic potential in the pathogenesis of RA [120].

Aside from the production of degradative enzymes and the initiation of the cytokine and chemokine cascades for inflammation, the release of NETs containing extracellular fibers, chromatin, cytoplasmic proteins and granule enzymes from neutrophils provides a great source for autoantigens driving autoimmune diseases [120]. Just as Khandpur and others discovered that NETosis was enhanced in neutrophils in RA synovial fluid and systemic circulation, sera extracted from RA patients, particularly those rich in ACPAs, were shown to significantly stimulate NETosis [101]. Appealingly, a therapeutic ACPA design to alter the binding of ACPAs, by targeting citrulline at a specific position, was documented to suppress NETosis from human neutrophils triggered by disease-relevant stimuli [121]. The changes in the neutrophil response upon ACPA alteration again strengthen the importance of ACPAs in triggering NETosis [121].

NETs have been demonstrated to augment inflammatory responses in RA synovial fibroblasts, including the induction of IL-6, IL-8, chemokines and adhesion molecules [101]. Additionally, the internalization of NETs via the receptor for advanced glycosylation end products—the TLR9 pathway in synovial fibroblasts—promotes an inflammatory phenotype and the upregulation of human HLA class II [104]. While the release of NETs is a source for citrullinated autoantigens, NETosis also promotes the citrullination of proteins within the synovial space. This resulted in the autoreactivity and affinity maturation of synovial B cells. Indeed, tissue-resident B cells within the ectopic lymphoid structures were also noted to generate high-affinity ACPAs targeting NETs [122]. The accelerated activity of PADs and externalized citrullinated autoantigens upon neutrophil netting likely further fuel the production of ACPAs [101,123]. These results, together with the ability of ACPAs to promote neutrophil netting, suggest a self-perpetuating cycle that can result in profound autoimmune reactivity.

#### 3.2.3. ACPAs Activate Complement via the Classical and Alternative Pathways

Many studies have validated the importance of complement activation in developing experimental antibody-mediated arthritis [124]. While RF plays a critical role in activating the complement system in RA, the role of ACPAs in complement fixation, specifically in the in vivo setting, remains controversial [125]. The in vitro data provided by Trouw suggests that ACPAs from patients with RA activate complement via the classical pathway in a dose-dependent manner. The alternative pathway is also involved in complement activation and sequential inflammatory processes, including the release of complement 5a (C5a) and membrane attack complex (MAC) formation [33]. The release of C5a upon complement cascade activation can lead to the chemotaxis of inflammatory cells, can increase vascular permeability, and can promote phagocytosis and the release of proinflammatory cytokines and chemokines [126]. It also amplifies inflammation and induces tissue injury via the release of free radicals and upregulates FcγRIIIa to enhance the interactions between ICs and cells [127,128]. MAC formation, on the other hand, leads to necrotic or apoptotic cell death. This not only results in tissue injury but also boosts further citrullination [100,102]. As the data suggest that the incorporation of IgM or IgA RF into ACPA-forming ICs triggers the activation of complement cascades, the presence of classical pentameric IgM RF additionally makes the process more efficient [33,72].

#### 3.2.4. Autoreactive B Cells Baring Surface ACPAs Promote T Cell Differentiation and Secrete Proinflammatory Cytokines

Aside from antibody production, autoreactive B cells that escape central and peripheral tolerance have also been shown to serve as APCs in promoting T cell maturation and differentiation into memory cells [129]. They play a critical role in the support of germinal-center-like structures (tertiary lymphoid tissues) within the inflamed synovium, and continuously fuel the autoimmune process [130]. Indeed, a proportion of B cells extracted from the inflamed synovial tissue were found to react with citrullinated autoantigens [122,131,132]. The data also suggest that the depletion of B cells hinders the ability of T cells to produce interferon-gamma (IFN-γ) and interleukin (IL)-1 [133]. Moreover, citrulline-specific T cells isolated from RA patients have been shown to display predominately Th1 and Th17 phenotypes [134,135]. While Th1 cells activate macrophages to serve as APCs and are the source of various proinflammatory cytokines, such as IFN-γ, IL-2 and TNF-α [136,137], Th17 cells not only stimulate the production of proinflammatory cytokines, chemokines and matrix metalloproteinases (MMPs) in RA synovial fibroblasts but also promote pannus growth, osteoclastogenesis and synovial angiogenesis via IL-17 production [138].

Additionally, subsets of memory B cells have been shown to express the receptor activator of nuclear factor kappa-Β ligand (RANKL) [139], a cytokine which is important for the regulation of bone homeostasis and osteoclastogenesis. Considering the ability of B cells to produce various proinflammatory cytokines [140], autoreactive B cells baring surface ACPAs critically support the progression of RA upon encountering their specific antigens; in this case, citrullinated peptides.

### 3.3. Interactions between ACPAs and Synovial Residential Cells

#### 3.3.1. Binding of ACPAs to form ICs via FcγR Promotes Osteoclast Differentiation and Proinflammatory Cytokine Production

The impact of ACPAs on osteoclasts was revealed by Harre and others upon their discovery of the strong association between ACPAs and osteoclast-mediated bone resorption markers in RA. By purifying ACPAs from patients with RA, they demonstrated that polyclonal ACPAs were capable of promoting osteoclast activation and differentiation, resulting in osteopenia and erosions in bones [141]. Considering that the effect of ACPAs can be mediated either through the direct binding of Fab to antigens or through Fc receptor signaling, efforts have been made to clarify the underlying mechanisms and validate ACPA-mediated osteoclast activities in RA-associated symptoms.

In the past decade, several reports have displayed exciting results explaining the two prominent features of RA manifestation, joint pain and bone erosion, via direct recognition and binding of the ACPA variable region to cellular targets on osteoclasts [142,143]. Utilizing experimental murine arthritis models, studies by Wigerblad and Krishnamurthy demonstrated that ACPAs enriched from human IgG can induce pain through the release of IL-8 and promote osteoclast differentiation in a IL-8-dependent manner [142,143]. While the significance of ACPA Fab-specific binding was demonstrated by the osteoclastogenesis induced by monomeric Fab fragments created from the ACPA variable domain independent of the constant region [142,143], it caught everyone by surprise, as the following report suggested that there was in fact no binding of monoclonal ACPAs to citrullinated antigens [144,145,146]. Aside from the dilemma regarding the monoclonal antibody binding specificity [147,148], Sun and others reported that the binding of osteoclasts and synovial fibroblasts by ACPAs from different clonalities exhibits distinct cellular effects, including increasing osteoclast differentiation and promoting synovial fibroblast migration, suggesting that antibodies with different clonality can function differently [149].

Similar to macrophages and dendritic cells, osteoclasts also contain sufficient amounts of FcγR [150]. FcRs in association with other immunomodulatory proteins on the osteoclast surface are critical for the development and function of osteoclasts [151]. As ICs containing IgG with various degrees of N-glycosylation are known to alter the differentiation of osteoclast precursors and cell activation [152,153,154], the glycosylation of the ACPA Fc fragment has been shown to enhance its affinity toward FcγR and increase its capacity to induce osteoclast activation and bone erosion [154]. This effect of ACPA-related osteoclast activation is further amplified in the concomitant presence of RF, as supported by the radiographic evidence [155,156].

#### 3.3.2. ACPAs Interact with Cellular Citrullinated Antigens and Promote Synovial Fibroblast Migration

Synovial fibroblasts contribute to the joint architecture through the formation of the intimal lining in the synovial anatomical space [157]. Although the signature architectural distribution allows synovial fibroblasts to be exposed to many disease-specific immune mediators, its interaction with ACPAs has seldom been discussed. As discussed earlier, it has been reported that the binding of synovial fibroblasts by distinct ACPAs can lead to synovial fibroblast migration via a phosphoinositide 3-kinase-mediated mechanism [149]. Recently, Corsiero and others identified a synovial B cell-derived RA recombinant monoclonal antibody capable of reacting with citrullinated fibroblast-derived calreticulin [158]. While the pathogenic role of the latter antibody awaits further elucidation, citrullinated peptides within synovial fibroblasts likely serve as neoantigens for ACPA variable domain targeting.

#### 3.3.3. ACPAs Induce Joint Destruction by Cross-Reacting with Type II Collagen Fibers

As chondrocytes are the main cellular component of articular cartilage, secreted extracellular matrix components composed of water, type II collagen, proteoglycans, non-collagenous proteins and glycoproteins make up the majority of the structure [159]. In the past, autoantibodies interacting with type II collagen in RA patients have been shown to bind with the cartilage components and antigens presented on the articular cartilage surface [160]. Recently, ACPAs were documented to cross-react with type II collagen, resulting in proteoglycan depletion and severe arthritis [54]. Although the pathogenic mechanisms were not discussed in the study [54], considering that complement activation has been shown in the antibody–cartilage surface interactions among RA patients [161], perhaps the binding of ACPAs with type II collagen induces joint inflammation and structural damage by triggering the complement cascade.

## 4. Potential of Therapeutic Approaches Targeting ACPAs for Patients with RA

Despite the clinical significance of RF and ACPAs and the current knowledge about their immunopathogenesis, no tailored treatments are specifically recommended for seropositive RA patients to date, nor for those specifically with high ACPAs [162]. Aside from the promising mechanisms discussed above, the plausibility of targeting ACPAs in RA treatment relies on the answers to the following questions. First, why is the expression of ACPAs not synchronized with the activity of RA? Accumulating evidence has also shown that the levels of ACPAs may not be ideal biomarkers to monitor the efficacy of methotrexate and TNF inhibitors in RA [20,21]. Even though rituximab and abatacept have been shown to control disease activity, the reduction in the level of ACPAs is very limited [163,164]. Moreover, upon the successful initial response to rituximab treatment, ACPAs do not increase in level prior to or during relapse following initial response [165]. Next, why does the disease not transfer along with placental transmission of antibodies during pregnancy? Although adoptive transfer of ACPAs has been demonstrated to induce arthritis in murine models [141], such a phenomenon has not been documented in humans.

**Figure 2:**
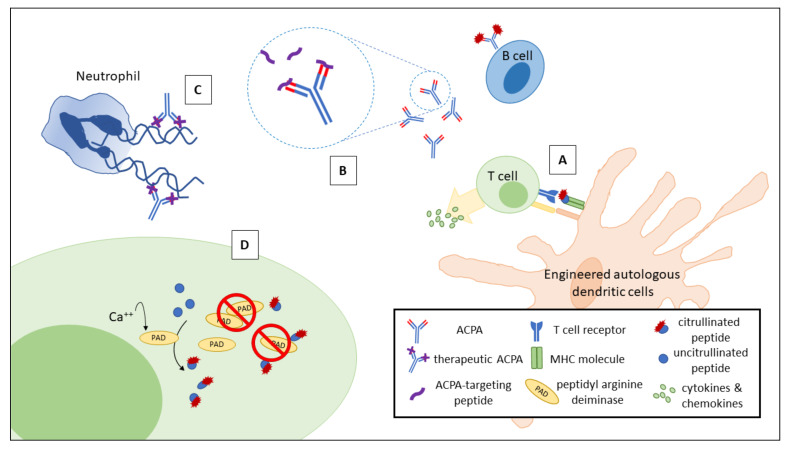
Potential treatment strategies for ACPA-mediated RA. Citrullinated peptide antigen-loaded autologous dendritic cells modified with an NF-κB inhibitor increase the ratio of regulatory to effector T cells (**A**). In (**B**), ACPA-targeting peptides derived from the fibrinogen α chain are capable of disrupting ACPA binding to its citrullinated antigens. Therapeutic ACPAs designed to interact with specific citrullinated residues can suppress NETosis and its relevant diseases (**C**). PAD inhibitors attenuate citrullination and reduce the generation of citrullinated neoantigens for ACPA targeting (**D**). ACPA, anti-citrullinated peptide antibody; RA, rheumatoid arthritis; PAD, peptidyl arginine deiminase; NF-κB, nuclear factor kappaΒ.

Although there are no differences in C3 and C4 levels in ACPA-positive and ACPA-negative RA patients, given that complement is activated by ACPA and studies suggest a pro-inflammatory role of C3 in the example of anti-TNF therapy, targeting ACPA may provide potential therapeutic benefit for RA patients [125]. While the levels of ACPAs may not correlate with the severity of overall joint symptoms [166], given their pathogenic and therapeutic potential, scientists are continuously investigating new methods targeting and reducing ACPA levels. As illustrated in Figure 2, antigen-specific tolerance strategies have been successfully documented by Benham and others recently [167]. Specifically, autologous dendritic cells modified with an NF-κB inhibitor were exposed to four citrullinated peptide antigens before being injected. Patients receiving such treatment showed an increased ratio of regulatory to effector T cells, a reduction in inflammatory cytokines and a decrease in disease activity 1 month after the procedure [167]. Furthermore, based on the success of anti-double-stranded DNA antibodies targeting peptides in systemic lupus erythematosus [168,169,170], scientists have identified two peptides derived from the fibrinogen α chain capable of disrupting ACPA binding to anti-cyclic citrullinated peptide (CCP2) [171]. Through grafting the citrullinated epitopes into a stable scaffold, the synthetic high-affinity peptide-based scavenger of ACPAs demonstrated superior stability with apparent low nanomolar affinity, which makes in vivo testing possible in the future [172]. Recently, therapeutic ACPAs specifically created to target citrulline residues in the N-terminus of histones 2A and 4 have been tested in various NET-relevant diseases, including inflammatory arthritis [121]. Although not specifically designed to treat diseases resulting from ACPA overproduction, the attenuation of NETosis and sequential inflammatory processes via citrulline blockade is a useful application referenced by the pathogenic mechanisms of ACPAs. Finally, considering the importance of citrullinated disorders preceding the generation of ACPAs, PAD inhibitors were also investigated in the treatment of ACPA-positive RA [173,174]. Recently, Willis and others reported that Cl-amidine, a pan-PAD irreversible inhibitor, partially inhibited joint inflammation in a murine arthritis model [173]. Since the safety of PAD inhibition may be a potential problem, novel compounds capable of inhibiting RA-specific, PAD4-induced citrullination are currently under investigation [174].

## 5. Conclusions

The present review summarizes recent progress on the characteristics of ACPAs and the possible biological effects of ACPAs on immune cells to elicit articular inflammation. As discussed, the spectrum of the isotypes, the maturation of their specificity, N-glycosylation and IC formation critically determine the immunopathogenicity of ACPAs. While ACPAs clearly demonstrate the potential to interact with responding cells and synovial tissues via IC formation and their agonistic activity, their role as a therapeutic target still requires more investigation. Perhaps strategies including altering antibody glycosylation [175], inducing ACPA competition with blocking aptamers, single-chain variable fragments or antibody Fab units [176], or inhibiting IC activation may be considered [177]. With the rapid advancement in the field of immunology and rheumatology, ACPAs may serve not only as a clinical marker but also as a therapeutic target for ACPA-positive RA patients with an erosive phenotype one day.

## Figures and Tables

**Table 1:** The distinct characteristics of ACPA, in comparison with RF [31].

	ACPA	RF
**Isotypes**	mainly IgG and IgA	IgM > IgG > IgA
**Clinical association**	specific for RA	common in various autoimmune diseases
**N-glycosylation**	extensive	limited
**Germinal center reactions**	repeated	limited
**Somatic hypermutations**	extensive	limited
**Class switching**	extensive	limited
**B cell activation**	T cell dependent	T cell dependent and/or T cell independent
**Producing plasma cells**	long lived plasma cells	short lived plasma cells and/or plasmablasts

## Abbreviations

Aa	Aggregatibacter actinomycetemcomitans
ACPA	anti-citrullinated protein antibody
APCs	antigen presenting cells
C5a	complement 5a
CCPs	cyclic citrullinated peptides
ERK	extracellular signal-regulated kinases
Fab	antigen-binding fragment
Fc	crystallizable fragment
FcγR	Fc gamma receptor
HLA	human leukocyte antigen
IC	immune complex
IFNγ	interferon-gamma
IL	Interleukin
JNK	c-Jun N-terminal kinase
MAC	membrane attack complex
MMPs	matrix metalloproteinases
NET	neutrophil extracellular trap
PAD	peptidyl arginine deiminase
P. gingivalis	Porphyromomas gingivalis
RA	rheumatoid arthritis
RF	rheumatoid factor
ROS	reactive oxygen species
SE	shared epitope
M-CSF	macrophage colony-stimulating factor
NF-κB	nuclear factor-kappaB
RANKL	receptor activator of nuclear factor kappa-Β ligand
TLR	Toll-like receptor
TNF-α	tumor necrosis factor-alpha
